# Paediatric Myelin Oligodendrocyte Glycoprotein Antibody-Associated Disease (MOGAD): A Case Report With Varied Clinical Manifestations

**DOI:** 10.7759/cureus.85173

**Published:** 2025-06-01

**Authors:** Nur Ain Mohamad, Safinaz Mohd Khialdin, Nur Shahirah Amir Hamzah

**Affiliations:** 1 Ophthalmology, Faculty of Medicine, Universiti Kebangsaan Malaysia Medical Centre, Kuala Lumpur, MYS

**Keywords:** acute disseminated encephalitis, bilateral, myelin oligodendrocyte gycoprotein, optic neuritis, paediatric

## Abstract

Myelin oligodendrocyte glycoprotein antibody‐associated disease (MOGAD) is an autoimmune demyelinating condition that can affect both the pediatric and adult populations. The initial phenotype for the pediatric group is different from the adult. Young children commonly present with acute disseminated encephalomyelitis followed by optic neuritis and/or transverse myelitis. Here we described the clinical presentation of a young girl diagnosed with MOGAD.

A three-year-old girl presented with an acute onset of reduced vision in both eyes preceded by neurological symptoms two weeks prior. Bilateral vision was profoundly reduced, visual acuity (VA) with Cardiff at 50cm in both eyes was 6/96 with positive reverse afferent pupillary defect grade 3 in the left eye. Examination showed bilateral grade one optic disc swelling with upper motor neuron signs. Magnetic Resonance Imaging (MRI) Brain findings were consistent with bilateral optic neuritis with multiple areas of hyperintense lesions with area of enhancement. MRI Spine revealed no spine involvement. Serology analysis showed positive anti-MOG antibody. The patient was treated with high-dose intravenous corticosteroid. Her vision improved subsequently.

There was limited knowledge on clinical phenotype and relapsing course in MOGAD, especially in the pediatric group. Knowing about variation in pediatric clinical presentation might guide ophthalmologists and pediatricians to reach an accurate diagnosis.

## Introduction

Myelin oligodendrocyte glycoprotein antibody‐associated disease (MOGAD) is an immune-mediated inflammatory demyelinating disease that targets the optic nerve, brain, and spinal cord [1]. It is characterized by distinct clinical and MRI features with serum or CSF fluid positive for myelin oligodendrocyte glycoprotein (MOG) antibody [1]. For the past few years researchers have established MOGAD as a distinct clinical entity. Although the clinical-MRI features might overlap with multiple sclerosis (MS), neuromyelitis optica spectrum disorder (NMOSD), and other central nervous system (CNS) demyelinating diseases, the International MOGAD Panel has recently published proposed criteria for the diagnosis of MOGAD [1]. The criteria proposed the presence of MOG-IgG as a core criterion, using MOG-IgG cell-based assays for diagnostic accuracy.

MOG was first detected in 1984 by a mouse monoclonal antibody raised against rat cerebellar glycoproteins [2]. It is a CNS-specific antigen located on the myelin sheath and oligodendrocytes outer surface [2], hence accessible to potential antibodies as compared to myelin basic protein (MBP) and myelin-associated glycoprotein (MAG) which is attached to the inner surface of cell membrane and found in the innermost layer of myelin sheets respectively [3]. It also acts as a marker of mature oligodendrocytes [3]. The main function of MOG is still poorly understood. However, it was suggested that MOG mediates interactions between myelin and the immune system and it does not play a direct role in myelination [4]. Osman et al. described the pathophysiology of MOGAD starting with T cell activation in peripheral blood by means of molecular mimicry, bystander activation, or MOG presentation in CNS-draining lymph nodes [3]. This was evidenced by presence of CD4 helper T cells that were dominantly found in lesion histology besides granulocytes, macrophages, microglia, and activated complements [3]. Following that, reactivation of T cells occurred in the subarachnoid and perivascular spaces by the antigen-presenting cells and inflammatory cells in the cerebrospinal fluid, which allows T cells to infiltrate the CSF parenchyma [5]. In CNS parenchyma, MOG IgG opsonizes MOG, activates the complements and antibody-dependent cellular cytotoxicity (ADCC), and induces intracellular signalling cascade causing demyelination in MOGAD [3,5,6].

Knowledge of variation in clinical presentation might assist clinicians in reaching a MOGAD diagnosis in the pediatric age group. MOG antibody testing should also be considered in all children presenting with CNS demyelinating disease. Here we describe a MOGAD case with initial presentation of acute disseminated encephalomyelitis.

## Case presentation

**Figure 1:**
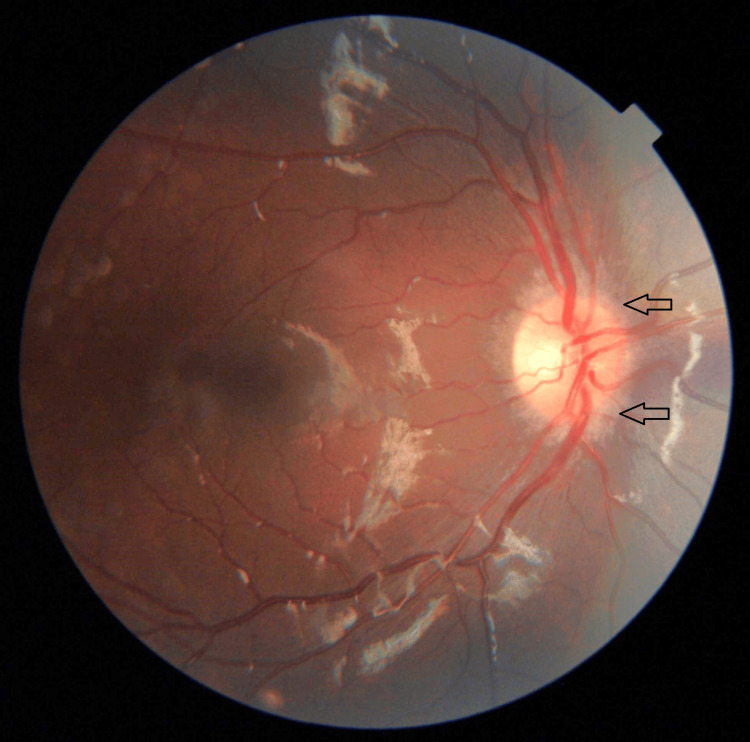
Grade one nasal optic disc swelling of the right eye (as shown by arrow).

**Figure 2:**
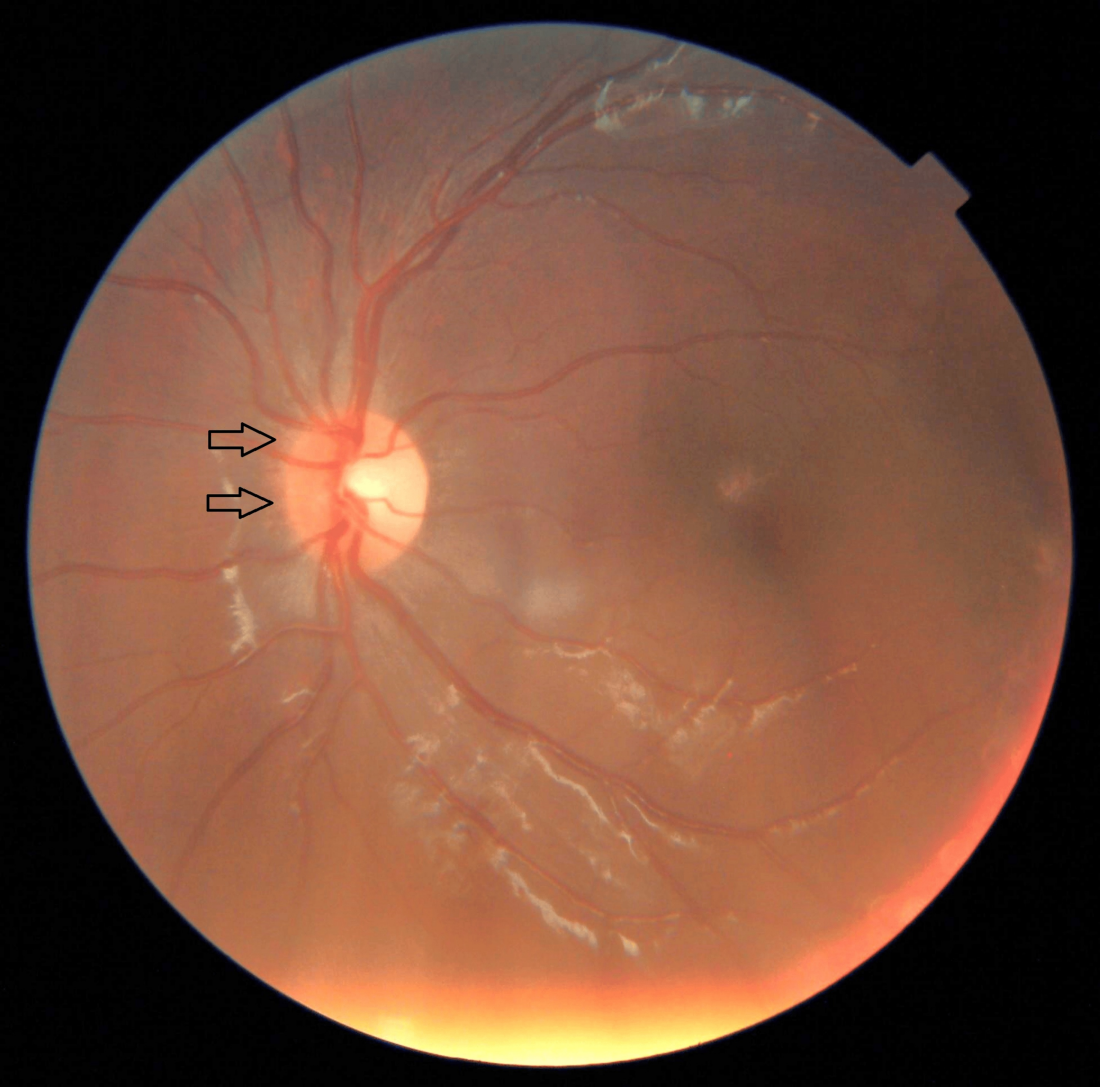
Grade one nasal optic disc swelling of the left eye (as shown by arrow).

**Figure 3:**
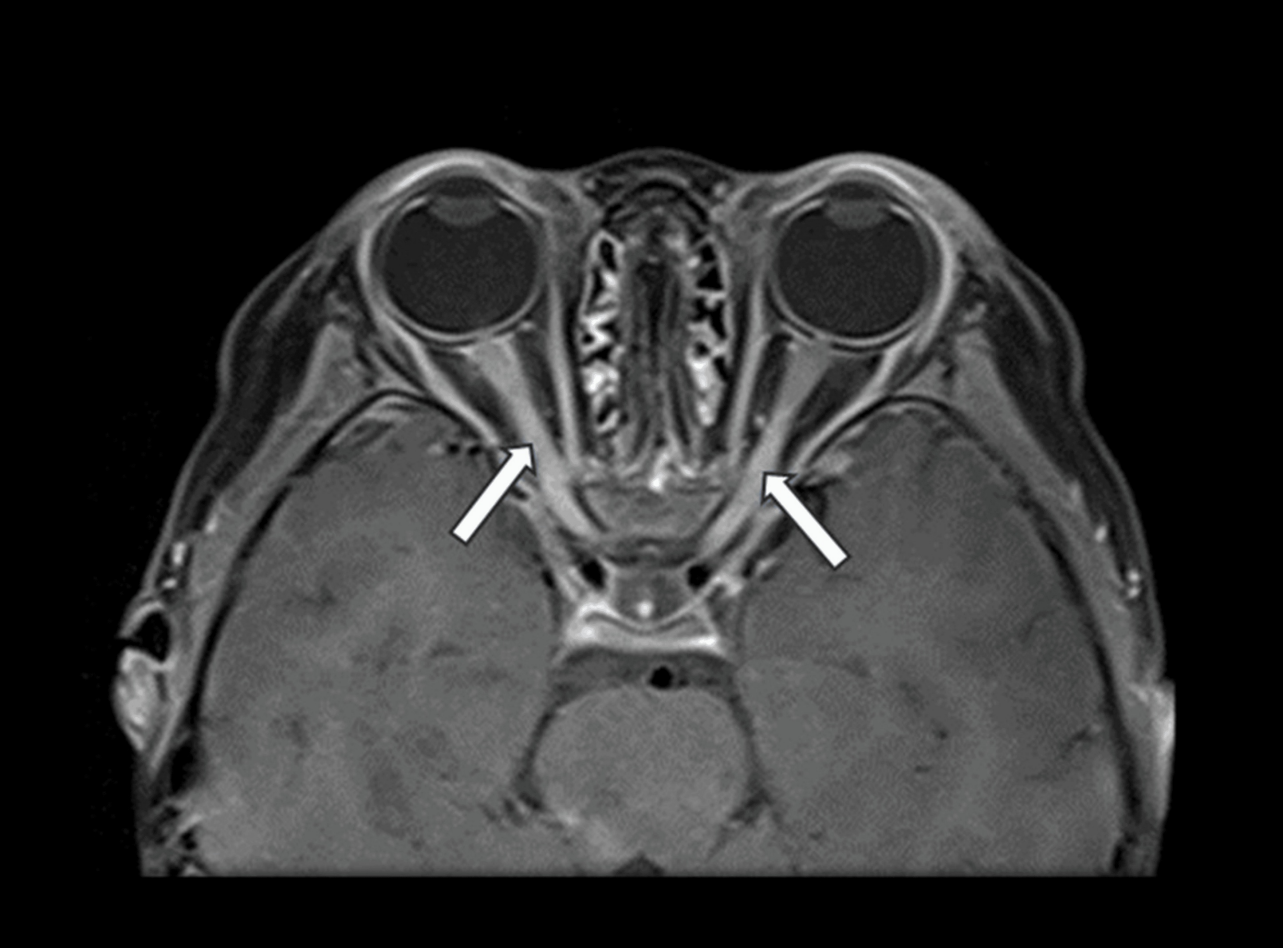
Bilateral optic nerve enhancement

**Figure 4:**
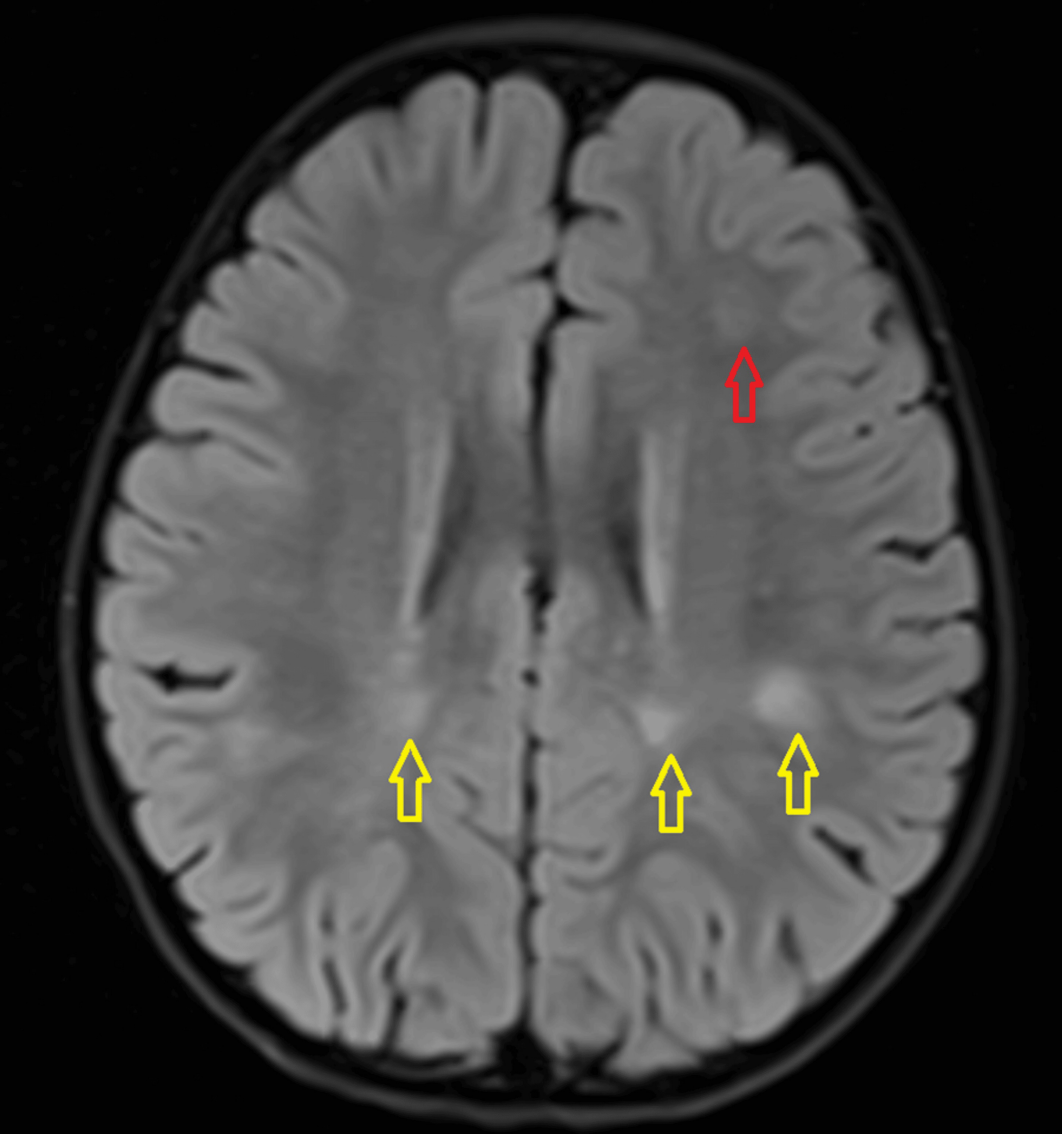
T2-Weighted Fluid-Attenuated Inversion Recovery (T2W/FLAIR) hyperintense white matter lesions noted at bilateral parietal lobes juxta-cortical region (yellow arrow) and at left frontal subcortical region (red arrow).

**Figure 5:**
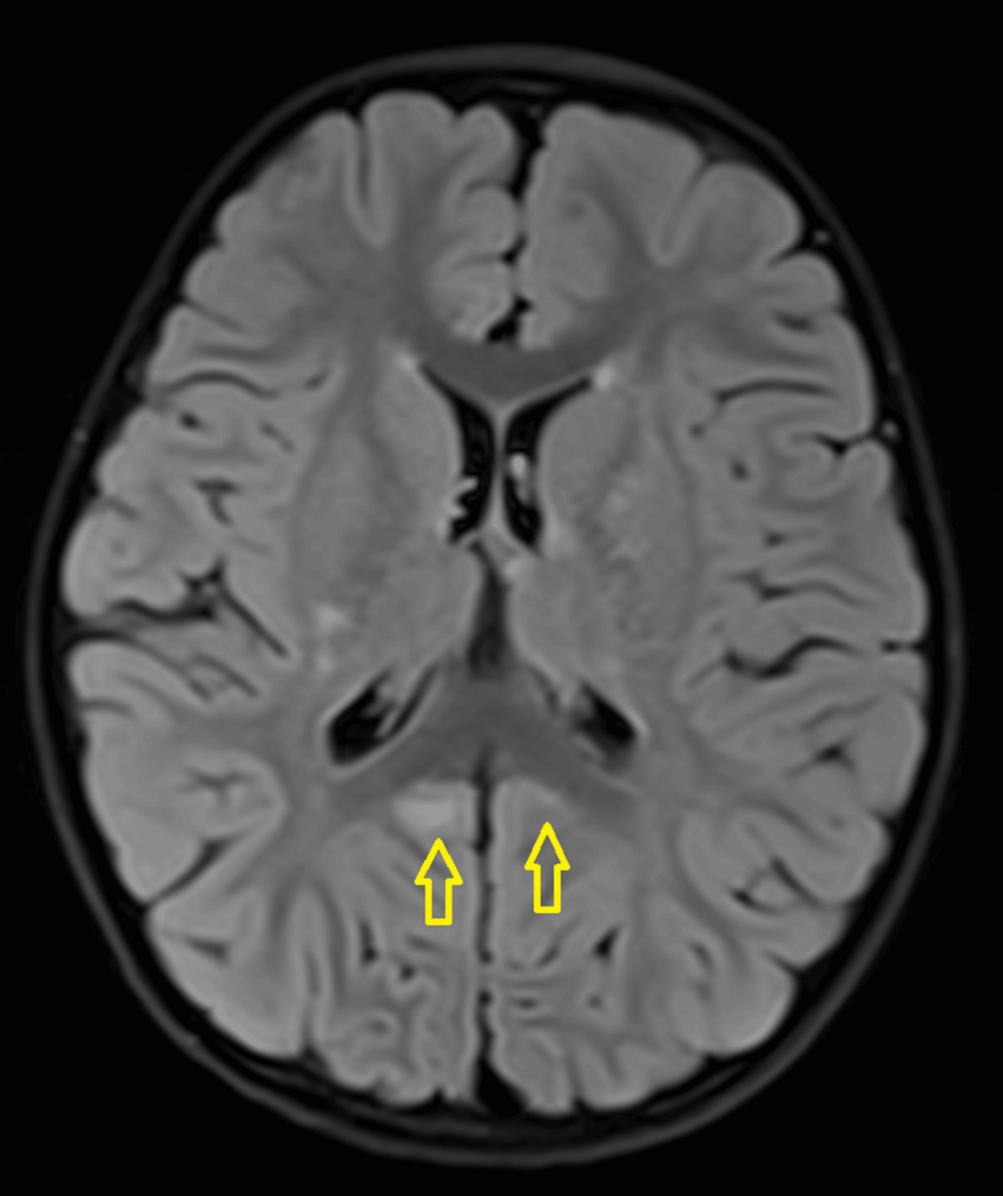
T2-Weighted Fluid-Attenuated Inversion Recovery (T2W/FLAIR) hyperintense white matter lesions noted at bilateral occipital juxta-cortical region (yellow arrow).

**Figure 6:**
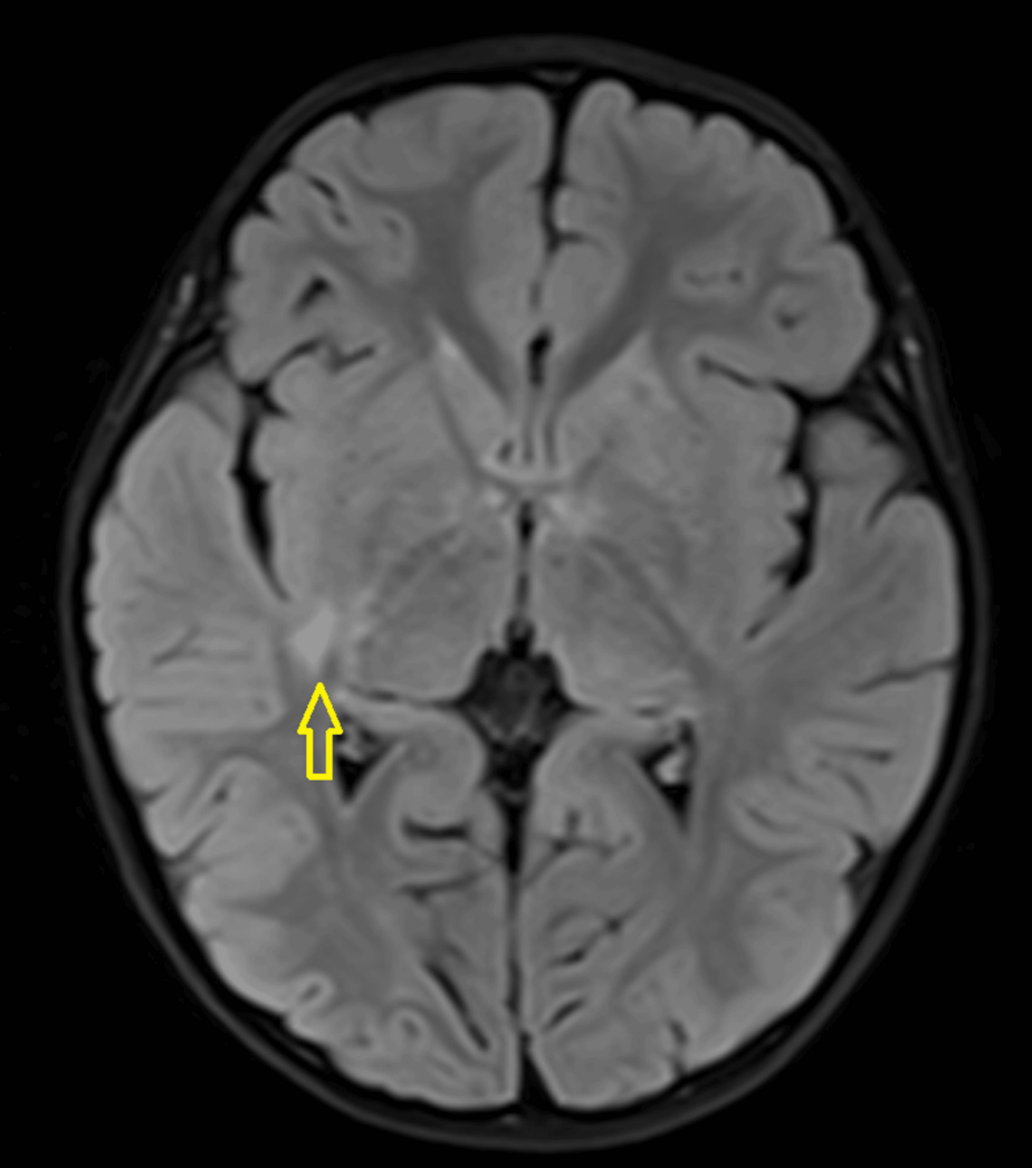
T2-Weighted Fluid-Attenuated Inversion Recovery (T2W/FLAIR) hyperintense white matter lesions noted at posterior limb of right internal capsule (yellow arrow).

**Figure 7:**
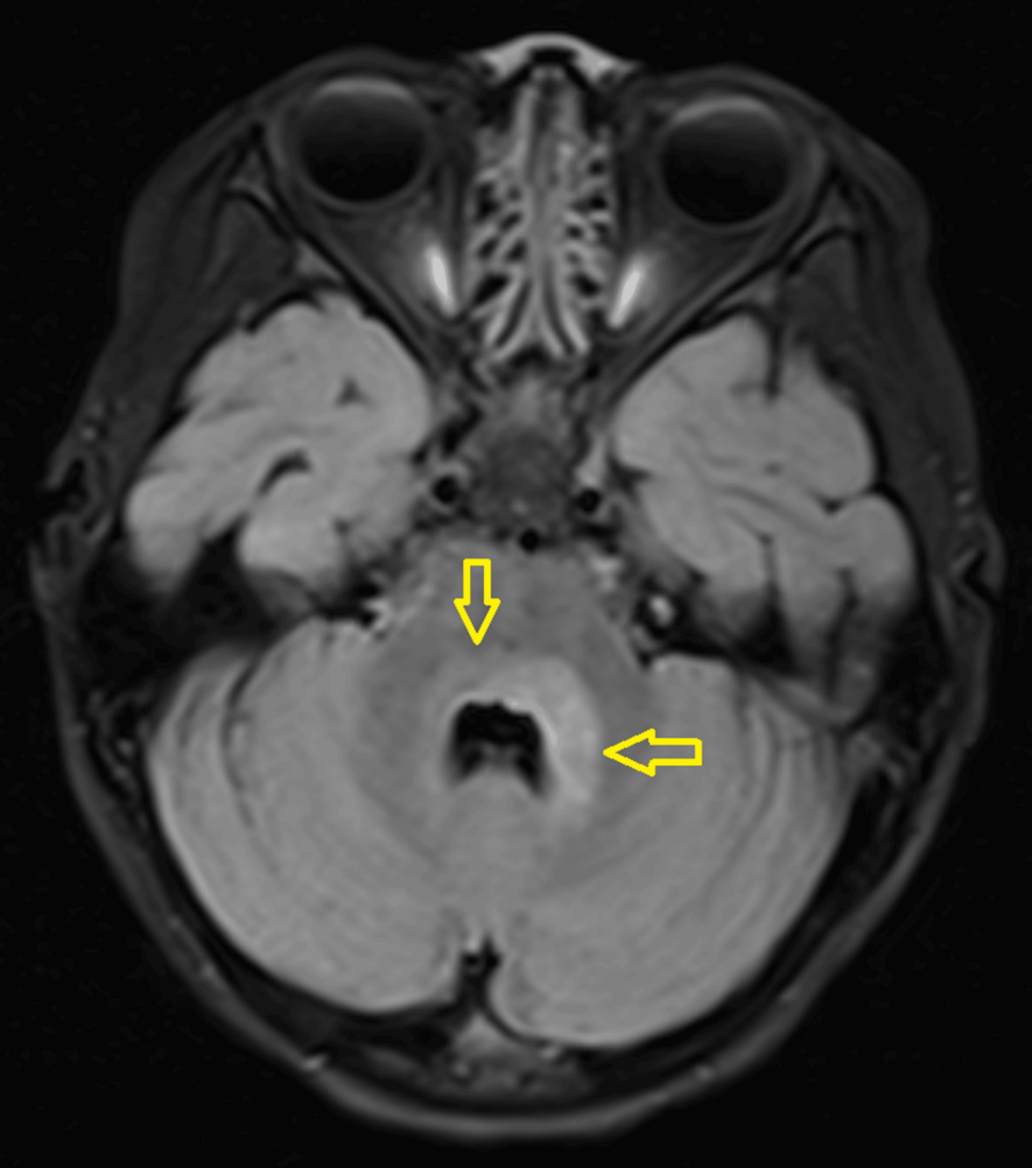
T2-Weighted Fluid-Attenuated Inversion Recovery (T2W/FLAIR) hyperintense white matter lesions noted at bilateral cerebral peduncles (yellow arrow).

**Figure 8:**
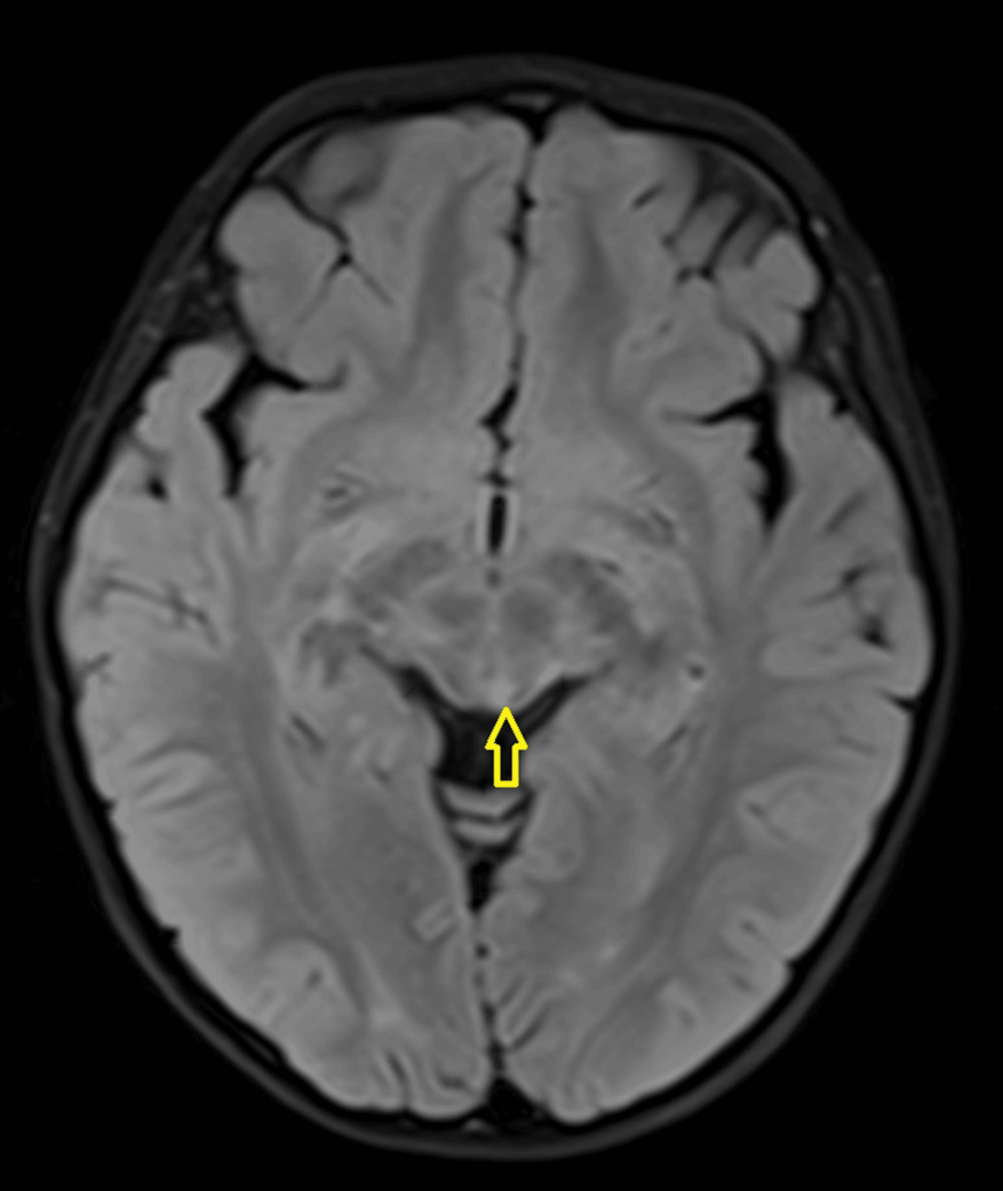
T2-Weighted Fluid-Attenuated Inversion Recovery (T2W/FLAIR) hyperintense white matter lesions noted at midbrain (yellow arrow).

**Figure 9:**
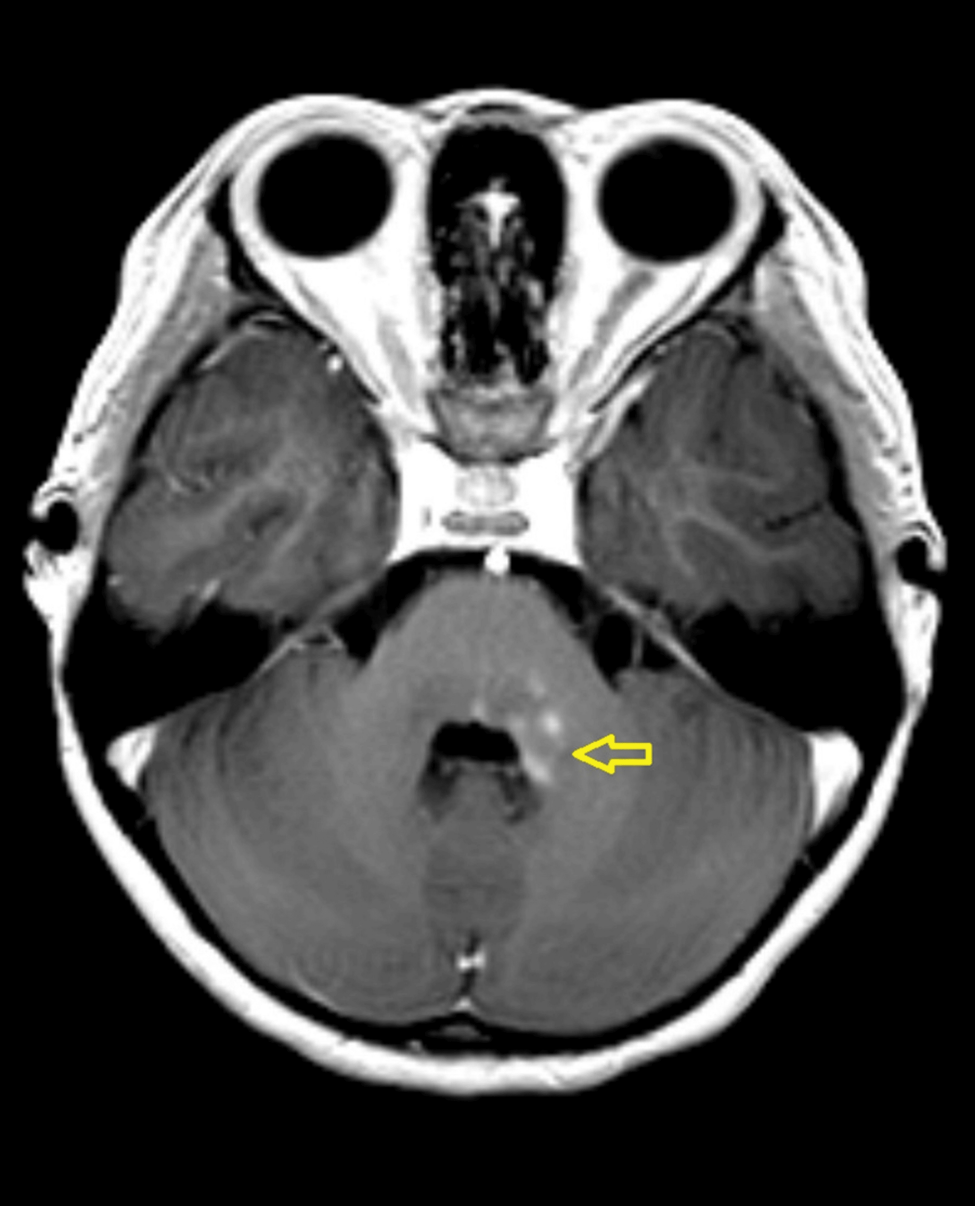
T1 post-contrast middle left cerebellar peduncle lesion (yellow arrow).

A three-year-old girl with no known medical illness presented with an acute onset of reduced vision in both eyes for four days with unstable gait, vomiting, and lethargy preceded by a prodromal viral illness three weeks prior. Parents noticed abnormal behavior, ataxic gait, lethargy, and vomiting a week after she had flu-like symptoms. She was investigated and treated as viral encephalitis and was well for a week before her parents noticed that she started to have poor vision in both eyes. Upon presentation, her vision was profoundly reduced bilaterally with visual acuity (VA) with Cardiff at 50cm in both eyes was 6/96 and a positive reverse afferent pupillary defect grade 3 in the left eye. Extraocular movements were intact in both eyes and she had no nystagmus. Fundus examination revealed grade one optic disc swelling in both eyes (Figures 1, 2). There were palpable, non-tender lymph nodes over the right cervical and right inguinal regions. Neurological examination revealed hypertonia and hyperreflexia of bilateral lower limbs with positive Babinski’s sign suggestive of upper motor neuron lesion. Magnetic resonance imaging (MRI) of the brain demonstrated findings consistent with bilateral optic neuritis (Figure 3). Multiple hyperintense lesions were observed on T2-weighted and Fluid-Attenuated Inversion Recovery (FLAIR) sequences, involving the juxtacortical white matter of the bilateral parietal lobes and left frontal subcortical region (Figure 4), bilateral occipital juxtacortical regions (Figure 5), the posterior limb of the right internal capsule (Figure 6), bilateral cerebral peduncles (Figure 7), and the midbrain (Figure 8). Post-contrast T1-weighted imaging revealed an enhancing lesion at the middle left cerebellar peduncle (Figure 9). Visual evoked potential (VEP) revealed delayed P100 latency of the left eye, which signifies anterior optic pathway dysfunction (Table 1).

**Table 1:** Visual Evoked Potential (VEP): Delayed P100 latency of the left eye which signifies anterior optic pathway dysfunction.

Run	Side	Label	N75	P100	N145	Amplitude
			ms	ms	ms	µV
R - VEP						
1.1	Right	O1 - Fz	66	110	150	3.5
1.2		Oz - Fz	76	111	154	7.0
1.3		O2 - Fz	77	110	154	5.0
2.1	Right	O1 - Fz	72	110	155	3.5
2.2		Oz - Fz	73	110	155	6.2
2.3		O2 - Fz	73	110	150	5.2
L - VEP						
1.1	Left	O1 - Fz	84	111	133	0.73
1.2		Oz - Fz	84	107	133	6.2
1.3		O2 - Fz	93	119	147	5.3
2.1	Left	O1 - Fz	83	118	143	0.22
2.2		Oz - Fz	83	118	146	6.1
2.3		O2 - Fz	91	117	148	3.9

A comprehensive panel of laboratory investigations was performed. CSF analysis including full examination (FEME), culture and sensitivity, and bacterial agglutination tests were unremarkable. Further CSF studies, including oligoclonal bands, aquaporin-4 antibodies, and IgG, IgA, and IgM levels, were all negative. In addition, serum antinuclear antibody (ANA), immunoglobulin levels, and cerebrospinal fluid oligoclonal bands were within normal limits. However, serology analysis showed positive anti-MOG antibody.

The patient was initiated on high-dose intravenous methylprednisolone at a dose of 30 mg/kg/day. A total of 15 doses were administered over five days, with close monitoring of clinical response and adverse effects. Following the seventh dose of intravenous methylprednisolone of 120 mg three times daily, there was a notable improvement in her vision. Cardiff visual acuity testing at 50 cm demonstrated right eye visual acuity (RVA) of 6/12 and left eye visual acuity (LVA) of 6/19. A relative afferent pupillary defect (RAPD) remained present in the left eye, graded as +1. Following completion of intravenous therapy, there was marked neurological improvement. Subsequently, the patient was transitioned to oral prednisolone with an initial dose of 15 mg with slow tapering dose over six months. The tapering regimen was well tolerated, and no steroid-related complications were reported. Throughout the treatment course, the patient was closely monitored for visual recovery, recurrence of neurological symptoms, and adverse effects of corticosteroids. Clinical and radiological follow-up did not reveal any evidence of disease relapse during or after corticosteroid discontinuation. By follow-up, visual acuity had improved to 3/3 bilaterally on Cardiff testing, reflecting near-complete recovery of vision. No relapse occurred during the 12 months of follow-up.

## Discussion

MOGAD can affect both adult and pediatric age groups, however, the incidence in the latter is much more common [7]. The prevalence of MOGAD worldwide is estimated at 1.3 to 2.5 per 100,000 with the annual incidence of 3.4-4.8 per million [8]. The prevalence is anticipated to rise in the next few years [1] following the proposed new diagnostic criteria by the International MOGAD Panel and easily available MOG antibody assays to ensure diagnostic accuracy. In Asia, Japan showed the highest incidence of MOGAD. The Japanese MOGAD survey showed an estimated prevalence of 1.34 per 100,000 population with an annual incidence of 3.9 per million [9], whilst Singapore estimated a prevalence of 1.26 per 100,000 population [10]. Comparing Japan and Singapore, Malaysia has a lower MOGAD prevalence of 0.12 per 100,000 population [11]. Both Singapore and Malaysia have a higher MOGAD proportion in the Indian ethnic group, suggesting potential influence of race [10,11].

Pediatric MOGAD can have variable clinical phenotypes and presentations. A systematic review by Santoro et al. reported that the most common presentation in the younger pediatric population of less than 11 years old was acute disseminated encephalomyelitis (ADEM) at 45%, followed by optic neuritis at 25% and transverse myelitis at 11% [12]. However, in pediatric populations older than 11 years, the most common presenting symptoms are optic neuritis at 58%, followed by ADEM at 13% and transverse myelitis at 10% [12]. Li et al. in his analysis of 93 children of median onset age six years who were diagnosed with MOGAD from Southern China also found that ADEM (58.1%) was the most common clinical phenotype [13]. However, there are isolated cases of MOGAD with atypical presentation. Maran et al. reported a paediatric MOGAD case who had idiopathic intracranial hypertension (IIH) as the initial presentation [14].

The diagnosis of ADEM must be made after exclusion of other etiologies and fulfillment of its diagnostic criteria. According to the International Paediatric Multiple Sclerosis Society Group (IPMSSG) Diagnostic Criteria for ADEM, 2013, all children diagnosed with ADEM must have the component of encephalopathy during the acute illness, combined with distinct characteristics of MRI lesions [15]. It has been explained that encephalopathy is characterized by sleepiness in 50%, irritability in 36%, obtundation in 20%, coma in 16%, and confusion in 8% of the patients [16]. Cole J et al. described several subtypes of ADEM including monophasic ADEM, multiphasic ADEM, ADEM-optic neuritis (ADEM-ON), and acute hemorrhagic encephalomyelitis (AHEM) [17]. The diagnosis of ADEM subtypes is made retrospectively following the course of the disease, taking into account the clinical and radiological features during clinic visits after the initial presentation. In this case study, the child developed profound bilateral eye blurring of vision three weeks after the onset of the neurological symptoms. Her MRI findings showed multiple supratentorial and infratentorial T2-weighted hyperintense lesion with enhancement with bilateral thickened optic nerves with homogenous enhancement along their segments with no spinal involvement. Serum MOG-Ab was positive. She met the definition criteria of ADEM-ON, which includes any episode of ADEM with one or more episodes of optic neuritis together with positive serum of MOG-antibody, patient fulfils the criteria of MOGAD. Wong et al. described 17 patients of less than 18 years old with ADEM-ON where MOG antibodies tested positive in 16 out of the 17 patients. He also described relapses in majority of his patients which were better controlled with corticosteroid therapy (prednisolone dose of more than 10mg per day) compared to steroid-sparing immunosuppressants [18].

Most patients who presented with ADEM are initially treated with broad-spectrum antibiotics and antiviral medications as the symptoms usually mimic acute CNS infection. However, once the diagnosis has been established, treatment is usually very specific. It is important to note that at the current moment, there are no published randomized controlled trials (RCTs) on MOGAD and demyelinating disease treatment trials in pediatric populations [19]. A systematic review described the use of intravenous methylprednisolone in 88%, followed by oral steroids in 67%, intravenous human immunoglobulin (IVIG) in 66% and plasma exchange in 33% for the treatment in the acute phase [20]. In acute setting for pediatric ADEM, high dose of intravenous glucocorticoids (10 to 30 mg/kg/day of methylprednisolone up to maximum dose of 1000 mg daily or 1 mg/kg/day dexamethasone with no clear maximum dose) will be administered in three to five days course followed by prolonged tapering dose of oral corticosteroid [17]. In relapsing disease, long-term maintenance treatment was either with prolonged oral corticosteroids in 53% of the studies, followed by azathioprine (51%), mycophenolate mofetil (45%), rituximab (41%) and periodic IVIG (26%) [17].

In terms of prognosis, 75-96% of pediatric MOGAD patients were reported to have a complete recovery. Pediatric MOGAD-ON were also shown to have better recovery compared to adults. In a study by Wendel et al., MOG-ON patients have complete recovery of visual acuity in 56-73% of patients [20]. Most pediatric MOGAD has monophasic disease and has good response to treatments. The long-term risk of relapse is approximately 35%, which is lower than adults.

## Conclusions

Pediatric MOGAD can have varied clinical presentation. Knowledge of the variation of presenting symptoms is the key for early diagnosis and treatment. Most pediatric MOGAD-ON patients have good response to treatments, complete recovery of visual acuity, and lower long-term risk of relapse compared to adults.
